# Comparing the expression patterns and differences of LOX and LOXL1 during the development of myodural bridge and ligamentum flavum

**DOI:** 10.3389/fcell.2026.1712643

**Published:** 2026-03-19

**Authors:** Yi-tong Sun, Zhao-ran Li, Hua-xun Lai, Wei Ma, Chan Li, Ji-hang Li, Yan-yan Chi, Sheng-bo Yu, Nan Zheng, Bo Liu, Jian-fei Zhang, Hong-jin Sui

**Affiliations:** 1 Department of Anatomy, College of Basic Medicine, Dalian Medical University, Dalian, Liaoning, China; 2 Dalian University Affiliated Xinhua Hospital, Dalian, Liaoning, China; 3 Department of Histology and Embryology, College of Basic Medicine, Dalian Medical University, Dalian, Liaoning, China

**Keywords:** development, ligamentum flavum, lysyl oxidase, lysyl oxidase like-1, myodural bridge

## Abstract

The ligamentum flavum (LF) is a passive stabilizing tissue that connects two adjacent vertebral arches and contributes to the enclosure the spinal canal, thereby closing the entire canal posteriorly. However, a gap exists at the posterior atlanto-occipital and atlanto-axial interspaces, where the LF is absent and replaced by the myodural bridge (MDB), which attaches to the spinal dura mater. The reasons for this anatomical difference and how it arises during development remain unclear. There are notable structural differences between MDB and LF. The MDB consists primarily of type I collagen fibers, while the LF contains both collagen and elastin fibers, with the latter comprising a higher proportion. This study speculates that LOX and LOXL1, as key regulators of fiber synthesis, play an essential role in the development of MDB and LF. Differences in the expression of LOX and LOXL1, which are involved in collagen and elastin cross-linking, may be one of the main factors underlying this structural divergence in the posterior cervical interspace. This study examined the cervicooccipital regions of rat embryos and young rats using histology, immunohistochemistry, and RT-qPCR to compare morphological and developmental differences between the MDB and LF. Additional experiments using the BAPN inhibitor were conducted to investigate the expression patterns and functional differences of LOX and LOXL1 during the development of the MDB and LF. Our aim is to clarify the developmental processes of the MDB and LF, identify the causes of structural differences in the posterior cervical interspaces, and reveal the molecular mechanisms regulating their formation. The conclusion of the current study as follows: ①The MDB and LF exhibit distinct histological developmental patterns: cells and fibers in the MDB align in an organized manner later than those in the LF, while the MDB forms its complete structures earlier than the LF. ②LOX and LOXL1 play critical roles in collagen and elastic fibers formation, mediating fiber synthesis in both the MDB and LF. ③Differential expression of LOX and LOXL1 during development leads to variations in fiber composition and maturation timing between the MDB and LF, contributing to their structural differences in the posterior cervical spinal interspace.

## 1 Introduction

The ligamentum flavum (LF) is a passive stabilizing tissue that connects two adjacent vertebral arches and participates in enclosing the spinal canal, thereby closing the entire canal posteriorly (Gillespie and Dickey, 2004). However, in the posterior atlanto-occipital and atlanto-axial interspace, a gap occurs where is not closed by the LF, but is crossed by the myodural bridge (MDB), which eventually attaches to the spinal dura mater. The reason why the MDB is present rather than the LF in the posterior atlanto-occipital and atlanto-axial interspace, and the origin of this difference in the posterior cervical interspace have not been clarified.

The MDB was first identified by Hack (Hac et al., 1995) in 1995, and its conceptual understanding has been progressively refined through ongoing research. The MDB is a connective tissue bridge that links the rectus capitis posterior minor, the rectus capitis posterior major, the oblique capitis inferior, the nuchal ligament, and the cervical dura mater located in the deep layer of the sub-occipital region. Its fibers originate from the ventral muscles and nuchal ligament within the deep layers of the sub-occipital region. They course obliquely downward, pass through the posterior atlanto-occipital and atlanto-axial spaces, and finally connect to the dura mater. With advances in MDB research, a new concept of “muscle-dura bridge complex” has been proposed, referring to the fusion and connection of fibers from various muscles and ligaments with the dura mater (Zheng et al., 2020). Studies have indicated the presence of MDB or MDB-like structures in mammals (Zheng et al., 2017), reptiles (Zhang et al., 2016; Zhaohuangfu et al., 2019) and birds (Dou et al., 2019; Okoye et al., 2018), with variations observed across species. For instance, the MDB structure in the finless porpoise is highly distinctive. It is a specialized muscle, referred to as the “occipital dura muscle” (Zhang et al., 2021); There are a large number of venous sinuses in penguin MDB (Chen et al., 2021). This indicates that MDB is a highly conserved structure with biodiversity, and accordingly, possesses multiple physiological functions. When the suboccipital muscles contract, pulling on the MDB helps prevent the dura mater from folding into the spinal canal (Scali et al., 2013) thereby protecting the spinal cord. In addition, the MDB may transmit proprioceptive signaling (Peck et al., 1984) and relate to cervicogenic headache (Hack and Hallgren, 2004). In recent years, some researchers have proposed the theory that MDB serves as one of the driving forces for cerebrospinal fluid circulation, Specifically, during head movement, contraction of the suboccipital muscles transmits tension through the MDB complex, displacing the dura mater and creating negative pressure within the subdural space, to facilitate cerebrospinal fluid flow (Sui, 2017).

In contrast to the MDB located at the craniocervical junction, the LF is situated in the intervertebral space below the axis. It assists in restoring the spine to its original position after flexion and extension (Gillespie and Dickey, 2004). The LF exhibits elastic properties: it elongates when stretched and returns to its resting length when the laminae approximate. Due to its elasticity, the LF does not form folds or protrude into the spinal canal.

There are significant compositional differences between MDB and LF. MDB is mainly composed of type I collagen fibers (Zheng et al., 2018). In contrast, the LF consists of both collagen and elastic fibers, with elastic fibers accounting for approximately 40%–60% of its composition (Nachemson and Evans, 1968; Ponseti, 1995). Collagen fibers and elastic fibers are formed through cross-linking of procollagen and tropoelastin, respectively, within the extracellular matrix. Lysyl oxidase (LOX) and lysyl oxidase-like protein-1 (LOXL1) are enzymes that catalyze the cross-linking of procollagen and tropoelastin, and are directly involved in determining the final structure of both the MDB and LF. These enzymes belong to a family of five isoforms that mainly differ in their pro-region sequences, which are critical not only for enzyme activation but also for substrate recognition and targeting to elastic fibers (Thomassin et al., 2005).

LOX is a copper-dependent monoamine oxidase, which is encoded and synthesized by the *Lox* gene. It is secreted by fibroblasts into extracellular space, where it oxidizes specific lysine residues within collagen or elastin to initiate covalent cross-linking, thereby forming collagen or elastin fibers (Smith-Mungo and Kagan, 1998). Importantly, the pro-region of LOX mediates its association with elastic fibers, and cleavage of the pro-region alters the physical properties of the mature enzyme, making it difficult to extract from tissues once bound to its substrate (Thomassin et al., 2005). LOX plays a vital role in maintaining the extracellular matrix stability and is essential for numerous biological processes. It is an indispensable for proper function of the cardiovascular, respiratory, and urinary systems. Deletion of the *Lox* gene results in perinatal mortality in mice (Mäki et al., 2002). Current studies also indicate that the LOX is highly associated with fibrotic diseases (Chen et al., 2019; Liu et al., 2016). In cancer research, LOX has been proved to promote tumor invasion and metastasis (Cox and erler, 2013). LOXL1 is homologous to LOX (Grau-Bové et al., 2015) and belongs to the same enzyme family. It specifically facilitates the cross-linking of elastin without interacting with collagen. Similar to LOX, the pro-region of LOXL1 is required for its deposition onto elastic fibers, and its mature form shows binding to the C-terminal half of tropoelastin (Thomassin et al., 2005). Some researchers hypothesize that LOXL1 may spatially guide the deposition of elastin (Liu et al., 2004). Knockout of the *Loxl1* gene leads to pelvic organ prolapse in postpartum mice (Li et al., 2021). Similar to LOX, elevated expression of LOXL1 is associated with conditions such as liver fibrosis (Zhao et al., 2018), pulmonary fibrosis (Tjin et al., 2017), tumors (Zeltz et al., 2019) and other diseases. LOX and LOXL isoenzymes differ from other copper amino oxidases in their quinone cofactor, the lysine tyrosylquinone (LTQ), which confers particular catalytic properties to these enzymes. β-Aminopropionitrile (BAPN) is a irreversible inhibitor of LOX activity that targets the active site of LOX and LOXL1 (Xia et al., 2019; Miana et al., 2015). Studies have shown that BAPN can suppress fiber synthesis and ameliorate organ fibrosis (Liu et al., 2016).

It is evident that LOX and LOXL1 play crucial roles in fiber synthesis, and their functions cannot be fully compensated by other regulatory factors involved in this process. The pro-regions of both enzymes are essential for directing their deposition onto elastic fibers by mediating interactions with tropoelastin, and the pro-form of the enzyme is what initially interacts with the matrix substrate (Thomassin et al., 2005). If LOX present in the MDB mediates the synthesis of both collagen and elastic fibers, it could lead to occlusion of the posterior atlanto-occipital and atlanto-axial spaces by the LF, thereby causing the loss of the distinctive MDB structure. In contrast, if LOX within the LF only mediates collagen fibers synthesis and LOXL1 is not expressed, the MDB may persist in the posterior cervical space outside the atlanto-occipital and -axis. Although the regulatory pathways of MDB and LF remain unclear, LOX and LOXL1 are situated downstream of fiber regulation (Miana et al., 2015), and directly reflect fiber synthesis activity. These enzymes are targeted to elastic fibers at the earliest stages of elastic fiber assembly and co-localize with elastin globules that coalesce into fibers (Thomassin et al., 2005). Investigating these enzymes can thus help infer the molecular regulatory mechanisms of MDB and LF through downstream signals. Therefore, this study speculated that LOX and LOXL1, as key regulators of fiber synthesis, play an indispensable role in the development of MDB and LF. Furthermore, the differential expression of LOX and LOXL1, which participate in the cross-linking of collagen and elastin respectively, may be a major factor contributing to structural differences within the posterior cervical space.

In this study, histology, immunohistochemistry, and RT-qPCR methods were employed to examine the cervico-occipital region in both rat embryos and young rats. Using embryonic and newborn SD rats, the morphological changes and differences between MDB and LF during development were compared. The neck of newborn rats were injected with the LOX inhibitor BAPN regionally, and the morphological changes in MDB and LF development were observed following the inhibition of LOX and LOXL1. This study aimed to explore the expression and differences roles of LOX and LOXL1 during the development of MDB and LF, investigate the developmental processes and potential causes of structural differences in the posterior cervical space, and to provide a foundation for elucidating the molecular regulatory mechanisms underlying MDB and LF development.

## 2 Materials and methods

### 2.1 Experimental animals

Sprague-Dawley (SD) rats were used in this study. To generate timed pregnancies, one male and one female rat were housed together overnight. The following morning, females were examined for the presence of a vaginal plug or sperm in a vaginal smear. If sperm was detected, the female rat was considered pregnant and recorded as E0, and was kept in a separate cage. Embryos were harvested at embryonic days 16–20 (E16-E20). The day of birth was designated as postnatal day 0 (P0). Postnatal rats were collected at P0, P7, P14, and P21.

### 2.2 Histological slices and staining

Embryos (E16-E20) were rinsed in phosphate-buffered saline (PBS) and fixed in 4% paraformaldehyde (PFA) for 24 h. Postnatal rats (P0, P7, P14, P21) were euthanized by intraperitoneal injection of tribromoethanol (0.005 mL/g) and transcardially perfused with warm saline, followed by 4% PFA. All samples were post-fixed in 4% PFA for 48 h. Postnatal specimens were decalcified in 10% ethylenediaminetetraacetic acid (EDTA) solution (approximately 5, 10, 15, and 20 days for P0, P7, P14, and P21 tissues, respectively). Subsequently, tissues were washed, dehydrated through a graded ethanol series, cleared in xylene, and embedded in paraffin. Sagittal sections (8 μm thickness) were obtained using a Leica HM450 rotary microtome. Sections were mounted on glass slides, rehydrated, and stained with hematoxylin and eosin (H&E) as well as Gomori’s aldehyde fuchsin. The preparation of the primary reagents in Gomori aldehyde fuchsin staining includes the following: (1) Gomori’s stain: Add 1 mL of glacial acetic acid to 100 mL of distilled water, then sequentially add 0.6 g of chromotrope 2R, 0.3 g of fast green FCF, 0.4 g of phosphotungstic acid, and 0.3 g of phosphomolybdic acid, stir until completely dissolved, filter, and store in a sealed container. (2) Aldehyde fuchsin stain: Add 1 g of basic fuchsin to 100 mL of 70% ethanol, stir until completely dissolved, then sequentially add 1 mL of concentrated hydrochloric acid and 2 mL of paraldehyde, let it stand at room temperature for 2–7 days to mature, with the maturity sign being the color change of the liquid from magenta to mauve; after maturation, filter it and store it in a sealed container in a 4 °C refrigerator, and take it out in advance to rewarm to room temperature before use. Histological analysis was performed using a Nikon research light microscope and an Olympus BH-2 polarizing microscope.

### 2.3 Immunohistochemical staining and quantitative analysis

Immunohistochemistry (IHC) was performed on paraffin sections to detect the expression of LOX in the MDB and of both LOX and LOXL1 in the LF. One-Step IHC Assay (DAB, compatible for Rabbit and Mouse) (KGC3201-60, KeyGen BioTech) was used for IHC staining. Anti-LOX antibody (ab174316, abcam) and LOXL1 Polyclonal Antibody HRP Conjugated (C91322HRP, SAB) were used as the primary antibody and diluted at a ratio of 1:400 with 1×PBS, followed by incubation at 4 °C overnight and rewarming at 37 °C for 30 min the next day. For quantitative analysis, the optical density of IHC staining was measured using ImageJ software. Ten non-overlapping, contiguous regions of interest (ROIs, 50 × 50 pixels each) were aligned parallel to the dura mater within the interface between the rectus capitis posterior minor muscle and the dura mater. Statistical analysis was conducted using SPSS software. Data were compared using one-way ANOVA followed by independent two-tailed Student’s t-tests. A P-value of less than 0.05 was considered statistically significant.

### 2.4 Intraperitoneal injection BAPN

To inhibit lysyl oxidase activity, rats received daily intraperitoneal injections of β-aminopropionitrile (BAPN) from P0. BAPN (#T13475) was purchased from Topscience (Shanghai, China). BAPN stock solution was prepared at a concentration of 500 mg/mL by dissolving 500 mg of BAPN in 1 mL of sterile normal saline (NS). A working solution of 10 mg/mL was freshly prepared by diluting 100 μL of the stock solution with NS to a final volume of 5 mL. The dosage was 0.05 g/kg from P0 to P7 and 0.1 g/kg from P8 to P13. The injection site was disinfected with 75% ethanol prior to each injection. Control animals received daily injections of an equal volume of normal saline, while a separate group received no treatment. These groups were designated as the BAPN, NS, and WT (wild-type) groups, respectively. All rats were sacrificed at P14, and tissue samples were collected for Masson’s trichrome staining.

### 2.5 Quantitative real-time PCR

Total RNA was extracted from 10 to 20 mg of tissue. Briefly, tissues were homogenized in Buffer RL with steel beads using a cryogenic homogenizer. cDNA was synthesized from 1 µg of total RNA. Quantitative real-time PCR (qPCR) was performed using SYBR Green master mix (Vazyme Biotech Co., Ltd.) on the real-time PCR system. The primer sequences used for amplification of the *Lox* gene were: forward CCT​GGA​TAT​GGC​ACC​GGT​TA and reverse GCG​GCT​TGG​TAA​GAA​GTC​AG. For the *Loxl1* gene: forward ACT​TGC​CTG​TGC​GAA​ACT​CT and reverse CCT​GCA​CGT​AGT​TGG​GAT​CT. For the *Gapdh* gene: forward TCC​AGT​ATG​ACT​CTA​CCC​ACG and reverse CAC​GAC​ATA​CTC​AGC​ACC​AG. Gene expression levels were normalized to Gapdh and analyzed using the 2(^-ΔΔCt^) method. Data visualization and statistical analysis were performed using GraphPad Prism 8.0.

## 3 Results

### 3.1 Morphological observations of MDB and LF

#### 3.1.1 HE staining

**FIGURE 1:**
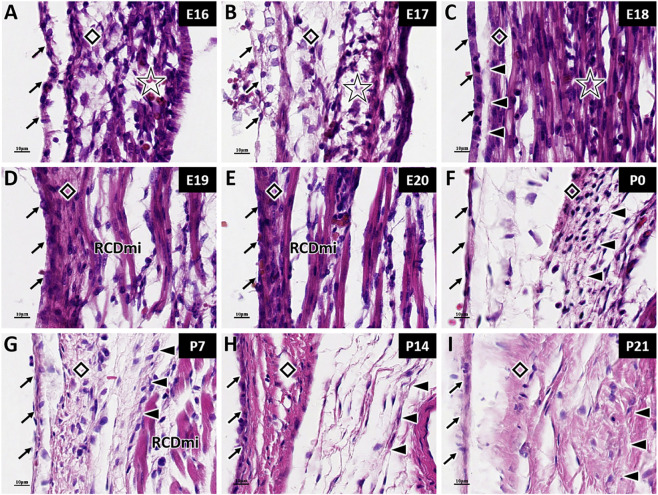
HE staining of myodural bridge fibers in rats at different developmental stages. RCDmi: Rectus capitis dorsalis minor muscle; →: Spinal dura mater; ◇: Posterior atlanto-occipital membrane; ☆: Occipital muscle region; ▲: Myodural bridge (MDB). Scale bar for panels **(A–I)** 10 μm. At E16 and E17 **(A,B)**, thin and sparse connective tissue is observed between the rectus capitis dorsalis minor muscle and the spinal dura mater. At E18 **(C)**, weak fibrous connections appear between the two structures. From E19 to E20 **(D,E)**, the muscle becomes tightly connected to the posterior atlanto-occipital membrane and spinal dura mater, with no visible fiber bundles. From P0 to P21 **(F–I)**, MDB fibers are clearly visible and gradually become denser with age.

At E16 and E17, thin and sparse connective tissues composed of by collagen fibers is observed in the atlanto-occipital space. This tissue contains numerous fibroblasts, which are irregularly arranged. At this stage, the rectus capitis major and rectus capitis minor on the dorsal aspect of the head are not fully developed and are difficult to distinguish. The overall muscle tissue is very thin, and the muscle fibers and their courses are not clearly defined. The MDB has not yet formed at this stage (Figures 1A,B). By E18, a weak fiber connection is observed between the rectus capitis minor muscle and the dura mater, indicating the initial formation of the MDB, though no distinct fiber bundles are apparent (Figure 1C). As embryonic development proceeds, the connection between muscles and dura mater becomes tighter, manifested as the muscle closely adhering to the posterior atlanto-occipital membrane and dura mater (Figures 1D,E).

At P0, the fibers from the rectus capitis minor on the dorsal side of the head can be clearly observed, traversing the posterior atlanto-occipital membrane and connecting to the dura mater. At this stage, the course of the MDB fiber is clearly visible, although the structure remain sparse overall. The fibers appear relatively straight with minimal curvature. Cells can be seen aligned along the fibers, indicating distinct directionality (Figure 1F). As the rats aged, the MDB fibers gradually became denser and thicker (Figures 1F–I). At P14, the cells within the fibers structures changed from oval to elongated spindle-shaped, indicating that maturation of fibroblasts, accompanied by a decrease in cells number (Figure 1H). At P21, the MDB fibers are noticeably denser, and the overall morphology closely resembled that of adult rats (Figure 1I).

**FIGURE 2:**
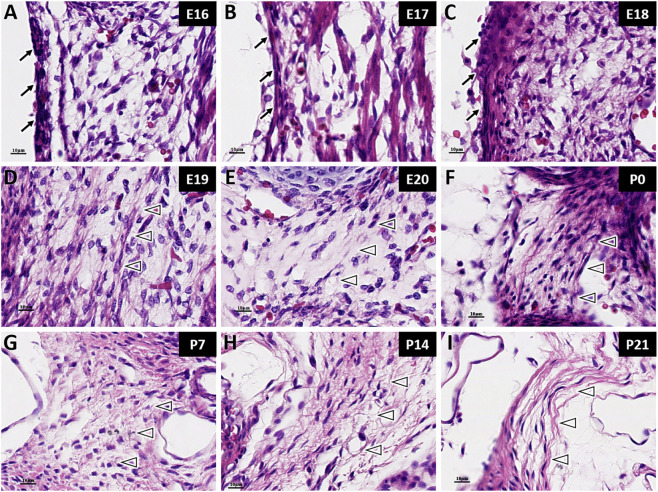
HE staining of ligamentum flavum fibers in rats at different developmental stages. →: Spinal dura mater; △: Ligamentum flavum (LF). Scale bar for panels **(A–I)** 10 μm. From E16 to E19 **(A–D)**, fibroblasts in the LF are disorganized, and fibers increase gradually. At E20 **(E)**, the fibers in the LF become clearly aligned, with cells arranged along the fiber direction. From P0 to P21 **(F–I)**, the fibers continue to increase and exhibit a wavy, crimped pattern.

At E16, the cells within LF are oval-shaped, and most are fibroblasts arranged irregularly. At this stage, fibers formation is minimal, scattered around the cells without clear bundling (Figure 2A). With embryonic development, the fibers gradually increase in quantity (Figures 2A–E). At E19, the arrangement of both fibers and cells became slightly more regular compared to earlier stages (Figure 2D). At E20, the fibers of the LF exhibited a clearly orientation, and the cells were aligned neatly along the direction of the fibers. However, the fibrous structure remained thin and loosely organized at this point (Figure 2E).

At P0, the fiber content of the LF had obviously increased, resulting in a more compact structure with an overall wavy appearance and emergence of distinct fiber bundles (Figure 2F). With advancing age, the fibers of the LF gradually became denser and thicker and were accompanied by an increase in curvature (Figures 2F–I). At P14, similar to the situation in MDB, fibroblasts had matured into fibrocytes, accompanied by a reduction in cells number (Figure 2H). At P21, the LF fibers appeared even denser (Figure 2I).

When comparing the fibers of MDB and LF, it was found that the LF exhibited organized fiber bundles at E20, with cells regularly along the fibers direction (Figure 2E). In contrast, the MDB lacked distinct fiber bundles during the embryonic period, which only became apparent at P0. After birth, the changes in fiber and cellular organization were significantly denser and more curled than those of the MDB.

#### 3.1.2 Gomori aldehyde fuchsin staining

**FIGURE 3:**
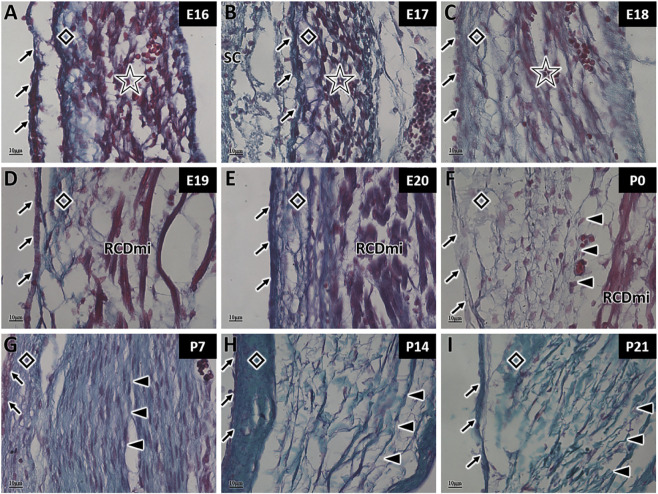
Gomori aldehyde fuchsin staining of myodural bridge fibers in rats at different developmental stages. SC: Spinal cord; RCDmi: Rectus capitis dorsalis minor muscle; →: Spinal dura mater; ◇: Posterior atlanto-occipital membrane; ☆: Occipital muscle region; ▲: Myodural bridge (MDB). Scale bar for panels **(A–I)** 10 μm. Collagen fibers are stained green, elastic fibers purple, and muscle fibers red. The MDB contains only green-stained collagen fibers, which increase in density during development **(A–I)**.

In Gomori aldehyde fuchsin staining, collagen fibers are stained green and elastic fibers are stained purple. During MDB development, the fibers were stained green, indicating the presence of collagen fibers at both embryonic and postnatal stages (Figures 3A–I). From E16 to E20, the green-stained area is minimal due to the unique structure of MDB during embryonic development (Figures 3A–E). The amount of green-stained collagen fibers gradually increase after birth (Figures 3F–I). This results confirm that the MDB contains only collagen fibers and lacks elastic fibers, distinguishing it from the LF.

**FIGURE 4:**
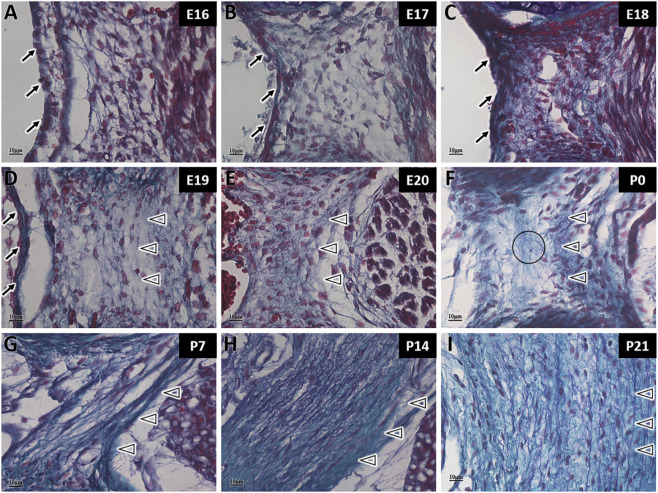
Gomori aldehyde fuchsin staining of ligamentum flavum fibers in rats at different developmental stages. →: Spinal dura mater; △: Ligamentum flavum (LF); ○: Scattered elastic fibers. Scale bar for panels **(A–I)** 10 μm. Collagen fibers are stained green, and elastic fibers purple. From E16 to E20 **(A–E)**, the LF contains only collagen fibers. At P0 **(F)**, scattered elastic fibers appear. From P7 onward **(G–I)**, elastic fibers form bundles and increase in number, interwoven with collagen fibers.

During the embryonic stage, the LF contains only green-stained collagen fibers, with no detectable elastic fibers present (Figures 4A–E). However, at P0, a small number of scattered purple-stained elastic fibers were observed, although their arrangement remained irregular at this stage (Figure 4F). At P7, distinct bundles of purple-stained elastic fibers became visible, interwoven with collagen fibers and exhibiting a more organized arrangement (Figure 4G). At P14 and P21, both elastic and collagen fibers in the LF had markedly increased, and their structure progressively approaches maturity (Figures 4H,I). The staining results indicated that collagen fibers in the rat LF were synthesized during the embryonic stage, while elastic fibers emerged after birth, demonstrating that collagen fiber development precedes that of elastic fibers.

### 3.2 Effects of LOX and LOXL1 on MDB and LF

#### 3.2.1 Masson staining

**FIGURE 5:**
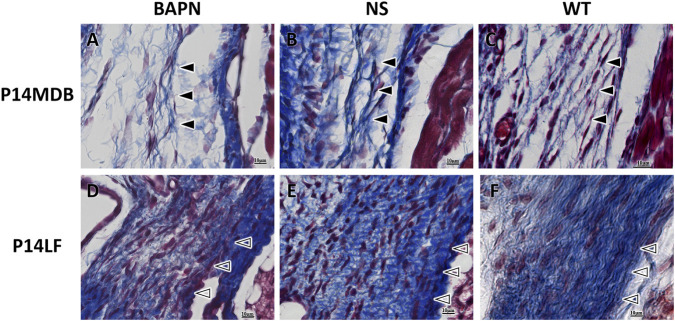
Masson staining of MDB and LF in different groups of rats at P14. ▲: Myodural bridge; △: Ligamentum flavum. Scale bar for panels **(A–F)** 10 μm. Fibers are stained blue, muscles red, and nuclei purple. No significant differences in fiber morphology are observed between the NS and WT groups **(B,C,E,F)**, representing the normal fiber state at P14. Compared to the NS and WT groups, the BAPN group shows reduced and sparse MDB fibers **(A)** and unevenly distributed LF fibers with gaps **(D)**.

After inhibition of LOX and LOXL1 with BAPN, Masson staining was performed at P14. In this staining, blue indicates fibers, red indicates muscle tissue, and purple indicates nuclei. In the wild-type group (WT group) and normal saline group (NS group), the MDB fibers were bundled, neatly arranged, and relatively dense (Figures 5B,C). The LF fibers were evenly distributed and generally highly dense (Figures 5E,F), representing the normal fibrous architecture at P14. In contrast, in the BAPN group, the MDB fibers were sparse and reduced in number (Figure 5A), while the LF fibers appeared slightly sparse and unevenly distributed, showing visible interstitial gaps (Figure 5D).

#### 3.2.2 RT-qPCR

**FIGURE 6:**
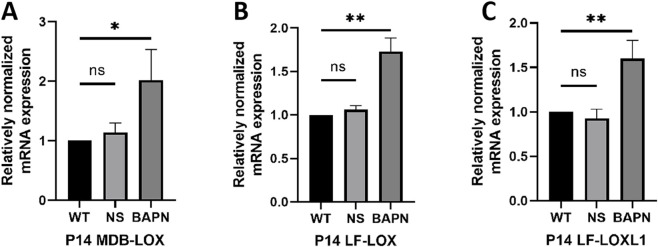
Comparison of Lox and Loxl1 expression in MDB and LF of different groups at P14. X-axis: Groups; Y-axis: Relative mRNA expression levels. At P14, compared to the WT and NS groups, the BAPN group shows increased Lox expression in the MDB **(A)** and increased Lox and Loxl1 expression in the LF **(B,C)**.

RT-qPCR analysis revealed that at P14, the expression of *Lox* in the MDB was significantly upregulated in the BAPN group compared with the WT and NS groups (Figure 6A). Similarly, the expression levels of *Lox* and *Loxl1* in LF were also increased in the BAPN group (Figures 6B,C). In contrast, no significant differences in expression of *Lox* and *Loxl1* were observed between the WT and NS groups (Figures 6A–C).

### 3.3 The expression of LOX and LOXL1 during the development of MDB and LF

#### 3.3.1 Immunohistochemical staining and quantitative analysis

In immunohistochemical staining, the target protein is visualized as brown staining, with brown areas in the sections indicating positive expression. The intensity of the brown color corresponds to the expression level of the protein—the stronger at the staining, the higher the expression level. The optical density of the immunohistochemically stained was quantified using ImageJ software. A higher optical density value reflects a greater expression level of the target protein.

##### 3.3.1.1 Expression changes of LOX in rat MDB at different stages

**FIGURE 7:**
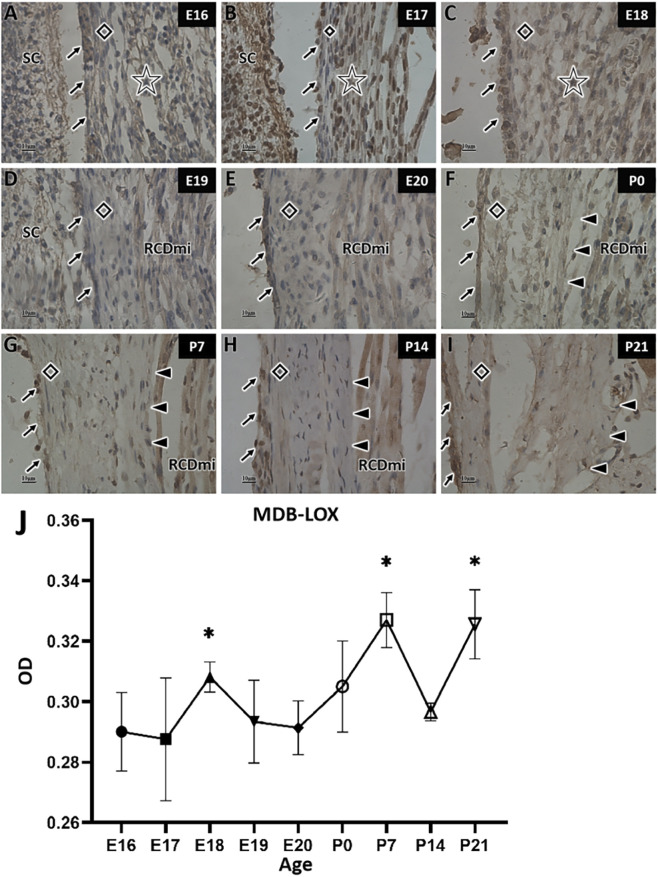
LOX immunohistochemical staining and quantitative analysis of myodural bridge fibers in rats at different developmental stages. SC: Spinal cord; RCDmi: Rectus capitis dorsalis minor muscle; →: Spinal dura mater; ◇: Posterior atlanto-occipital membrane; ☆: Occipital muscle region; ▲: Myodural bridge (MDB). Scale bar for panels **(A–I)** 10 μm. LOX staining is darker at E18, P7, and P21 **(C,G,I)**, lighter at E16, E17, E19, E20, and P14 **(A,B,D,E,H)**, and moderate at P0 **(F)**. Panel **(J)** shows LOX expression changes in the MDB over time, with the X-axis (Age) representing rat age and the Y-axis (OD) representing optical density values.

In the LOX staining of MDB fibers, the increases in LOX expression occurred intermittently rather than continuously. Strong staining of MDB fibers was observed at E18, P7 and P21 (Figures 7C,G,I), indicating high LOX expressed at these three stages. In contrast, at E16, E17, E19, E20 and P14, the brown staining in the fibers between the dura mater and muscles in the posterior occipital region, or those associated with the rectus capitis minor muscle, was very faint or nearly absent (Figures 7A,B,D,E,H), suggesting weak positive expression and low LOX levels during these times. At P0 (Figure 7F), the MDB fibers exhibited moderate staining, reflecting intermediate positive expression and moderate LOX expression at this stage.

Quantitative analysis revealed three distinct time points during MDB development at which LOX expression increased. During the embryonic stage, LOX expression in MDB was significantly higher at E18 than at E17, indicating a marked increase between these stages. However, no significant differences in LOX expression were observed during the remainder of the embryonic period. After birth, although no difference was observed between P7 and P21, but LOX expression at both stages was significantly higher than at most other stages. At P0 and P14, LOX expression did not differ significantly from that during the embryonic stage, nor was there a significant difference found between these two postnatal stages. Therefore, the LOX expression pattern during MDB development can be summarized as follows: low expression at E16 and E17, an increased at E18, stable expression from E19 to P0, a significant increase at P7, a decrease at P14, and renewed increase at P21 (Figure 7J).

##### 3.3.1.2 Expression changes of LOX in rat LF at different stages

**FIGURE 8:**
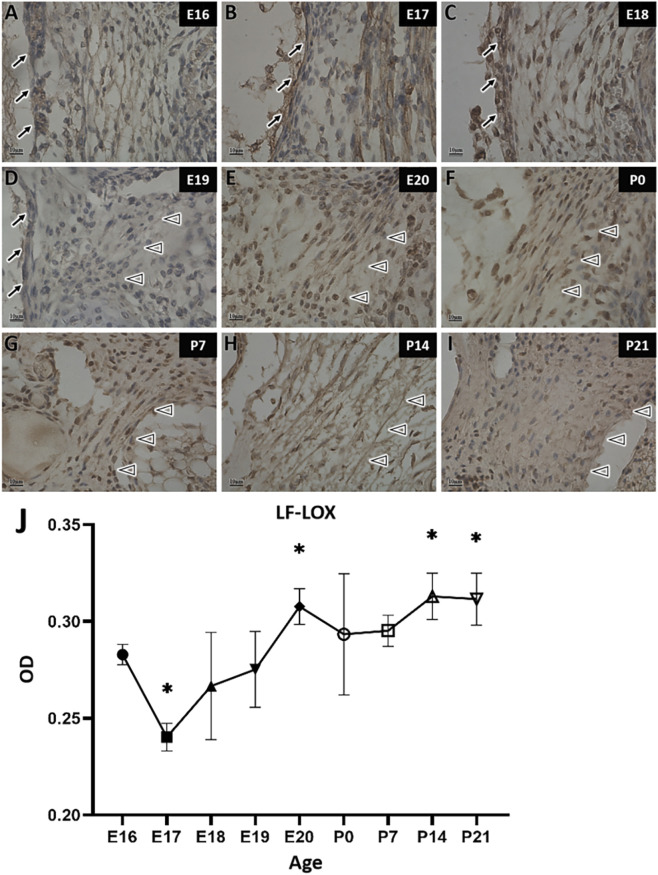
LOX immunohistochemical staining and quantitative analysis of ligamentum flavum fibers in rats at different developmental stages. →: Spinal dura mater; △: Ligamentum flavum. Scale bar for panels **(A–I)** 10 μm. LOX staining is darker from E20 to P21 **(E–I)** and lighter from E16 to E19 **(A–D)**. Panel **(J)** shows LOX expression changes in the LF over time, with the X-axis (Age) representing rat age and the Y-axis (OD) representing optical density values.

In the LOX staining of LF fibers, LOX expression showed a progressive increased, with E20 marking a transition between high and low expression periods. From E20 to P21, the fibers exhibited intense staining (Figures 8E–I), indicating high levels of LOX expression. Prior to E20, staining intensity showed minimal change (Figures 8A–D) and was noticeably weaker compared to the period from E20 onward.

Quantitative analysis revealed that LOX expression was lower at E17 than at all other stages except E18. LOX expression at E20 was differed significantly significantly from all embryonic stages except E16. No statistically significant difference was observed between any embryonic stage and the postnatal period. Similarly, no significant difference in LOX expression were detected across postnatal periods. However, LOX expression at P14 and P21 was significantly higher than during the embryonic stages, indicating a pronounced increase. In summary, throughout LF development, LOX expression decreased from E16 to E17, reaching a distinct trough at E17, rose slightly increased at E19, and then remained stable. At E20, LOX expression increased significantly and was maintained at high levels from birth through P21 (Figure 8J).

##### 3.3.1.3 Expression changes of LOXL1 in rat LF at different stages

**FIGURE 9:**
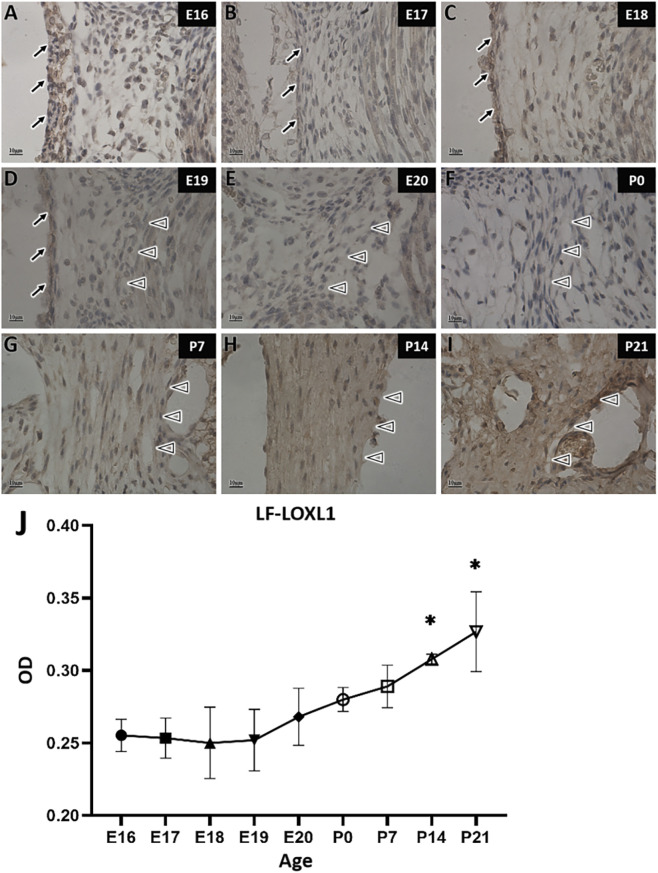
LOXL1 immunohistochemical staining and quantitative analysis of ligamentum flavum fibers in rats at different developmental stages. →: Spinal dura mater; △: Ligamentum flavum. Scale bar for panels **(A–I)** 10 μm LOXL1 staining is lighter before P0 **(A–F)** and darker after P0 **(G–I)**. Panel **(J)** shows LOXL1 expression changes in the LF over time, with the X-axis (Age) representing rat age and the Y-axis (OD) representing optical density values.

Immunostaining of LF fibers for LOXL1 revealed an expression pattern similar to that of LOX, with high expression during a continuous period. P0 served as the demarcation point between the high and low expression stages. Before and at P0 (Figures 9A–F), LF fibers were lightly stained indicating weak positive expression and low LOXL1 expression from embryonic day 16–20, as well as at birth. After P0, staining intensity increased (Figures 9G–I), showing strong positive expression and suggesting markedly elevated LOXL1 levels during the postnatal period.

Quantitative analysis revealed no significant changes in LOXL1 expression in LF during the embryonic period, with overall expression remaining low. However, postnatal LOXL1 expression differed from the embryonic period. LOXL1 expression at P0 and P7 was higher than at most embryonic stages. Specifically, at P0, LOXL1 expression was higher than at E17 to E19, and at P7, it was higher than at E16 to E19. Compared with all other time points, LOXL1 expression at P14 and P21 was significantly elevated, indicating a pronounced increase. Therefore, during LF development, LOXL1 expression remained largely unchanged and low level throughout the embryonic period. It increased postnatally compared to the embryonic period levels, with particularly marked elevation at P14 and P21, coinciding with the growth and development of the rats (Figure 9J).

##### 3.3.1.4 Differences in expression of LOX and LOXL1 in rat LF at different stages

**FIGURE 10:**
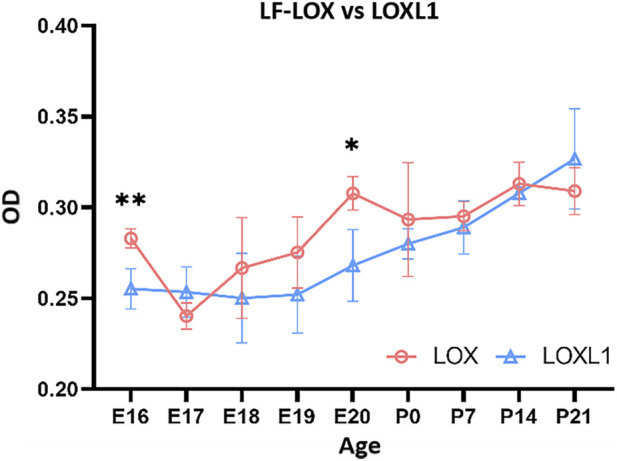
Comparison of LOX and LOXL1 expression in ligamentum flavum fibers at different developmental stages. X-axis (Age): Rat age; Y-axis (OD): Optical density values. LOX expression is slightly higher than LOXL1 overall.

The LOX expression in the LF was higher than that of LOXL1 at E16 and E20, while no significant differences were observed during other periods. Overall, LOX expression in LF was slightly higher than that of LOXL1. The expression patterns of LOX and LOXL1 in LF were somewhat similar, both showing low expression in the early stages and high expression in the later stages. The main difference between them was that LOX expression fluctuated during the embryonic stage, with a low point at E17. In contrast, LOXL1 expression remained relatively stable and consistently low throughout embryonic period, without significant fluctuation. LOX expression began to increase slightly as earlier at E20. Although LOXL1 also showed a slightly upward at this time, the change was not statistically significant, and its major increase occurred postnatally. Marked upregulation of LOXL1 was observed at P14 and P21. Although LOXL1 expression remained elevated after birth, it did not increase progressively over time but exhibited minor fluctuated within a certain range (Figure 10).

##### 3.3.1.5 Differences in LOX expression between MDB and LF in rats at the same time

**FIGURE 11:**
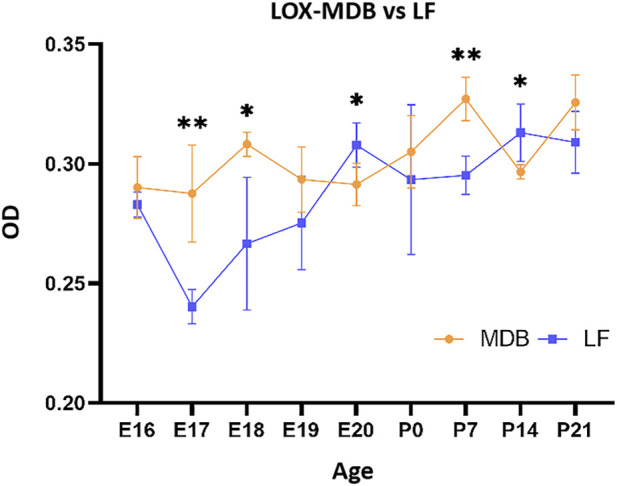
Comparison of LOX expression in myodural bridge and ligamentum flavum at different developmental stages. X-axis (Age): Rat age; Y-axis (OD): Optical density values. From E16 to E19, LOX expression is higher in the MDB than in the LF. At E20, LOX expression is higher in the LF. At P7, LOX expression is higher in the MDB, and at P14, it is higher in the LF.

During the embryonic stage, LOX expression in MDB was higher than that in LF from E16 to E19, indicating that LOX increased earlier in MDB compared to that in the LF. By E20, LOX expression in LF had increased and surpassed that in MDB. After birth, LOX expression in MDB gradually increased, becoming higher again than that in the LF at P7. At P14, LOX expression in the LF increased once again and surpassed the levels in MDB. After P21, no significant difference in LOX expression was observed between the two regions, and both remained high expression levels. Overall, the changes in LOX expression in the MDB and LF exhibited a largely complementary pattern. LOX expression in MDB showed more pronounced fluctuation, with peaks at E18, P7, and P21. In contrast, LOX expression in the LF was relatively stable, remaining low during early embryonic stages and increasing gradually from E20 onward (Figure 11).

#### 3.3.2 RT-qPCR

**FIGURE 12:**
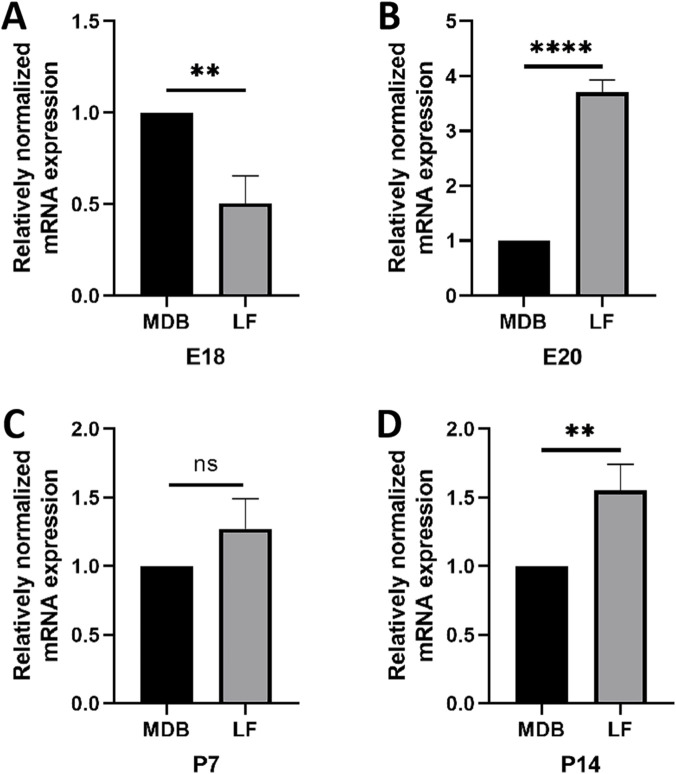
Comparison of Lox expression in MDB and LF at different developmental stages. X-axis: Groups; Y-axis: Relative mRNA expression levels. At E18, Lox expression is higher in the MDB than in the LF **(A)**. At E20, it is lower in the MDB **(B)**. At P7, there is no significant difference **(C)**. At P14, it is higher in the MDB **(D)**.

Based on the results of IHC, four stages of MDB (E18, E20, P7 and P14) were selected for RT-qPCR analysis. The results indicated that Lox expression in MDB was higher than that in LF at E18 (Figure 12A). At E20, the expression of Lox in MDB decreased and was lower than that in LF (Figure 12B). At P7, no significant difference in Lox expression was observed between MDB and LF (Figure 12C). At P14, Lox expression in MDB had increased again and was higher than that in LF (Figure 12D).

## 4 Discussion

The MDB and LF are both important and essential structures in the posterior cervical space. The MDB, a connective tissue bridge between the deep suboccipital muscles and the dura mater, facilitates the dura mater to displace through muscle contraction. This action helps prevent the dura mater from collapsing (Chen et al., 2021) and generate negative pressure in the subarachnoid space as a driving force to promote cerebrospinal fluid circulation (Hack and Hallgren, 2004). The LF, which connects adjacent vertebral arch plates and contributes to the formation of the spinal canal, primarily functions to maintain spinal stability. While no LF sealing the space between the occipital and posterior atlanto-axial regions, this area is traversed by the MDB. Both components are secreted by fibroblasts and synthesized in the extracellular matrix (Smith and Rennie, 2007; Kim et al., 2021), but in the atlanto-occipital and posterior atlanto-axial spaces, the MDB formed in place of LF. Previous studies have indicated that apoptosis is active in the occipital and posterior atlanto-axial spaces of rats between E12 and E16. It has been suggested that the MDB may regulate the connection between the suboccipital muscles and the dura mater, as well as collagen fiber production via apoptosis, thereby supporting its own morphogenesis (Song, 2021).

### 4.1 Developmental histological differences: MDB vs. LF

In this study, HE and Gomori aldehyde fuchsin staining were used to observe the changes in cells and fibers during the development of MDB and LF.

#### 4.1.1 Delayed formation and alignment of MDB

HE staining revealed MDB presence at E18. During embryonic development, the muscle clung to the posterior occipital membrane and dura mater, with clear fibers emerging after birth, consistent with prior reports (Lai et al., 2021). LF, however, was detected as early as E16. In human embryos, MDB appears later than LF. A myodural bridge-like structure extends through the atlanto-axial space connecting to the dura mater (Song et al., 2023), while LF (Misawa et al., 1994) is visible at 12 weeks. LF forms earlier and encloses the spinal canal, exclude the atlanto-occipital and posterior atlanto-axial spaces. This may allow space for MDB development, though the mechanism requires further investigation.

Additionally, during the development of MDB and LF, fiber and cells transition from scattered and disordered distribution to orderly alignment along the direction of mechanical stress. LF fibers become aligned at E20, while MDB fiber alignment appears at P0. Studies indicate that mechanical transduction occurs between cells and extracellular matrix (Monemian Esfahani et al., 2019). Without mechanical stress, cells remain irregularly arranged; once stress is applied, they align along its direction—a pattern also observed in fibers (Stopaka and Harris, 1982). Thus, the organized arrangement of cells and fibers in both MDB and LF is likely influenced by mechanical stress.

At E20, the cells and fibers of LF align orderly, likely due to the mechanical stress from vertebral arch development. It was shown in Lai’s study (Lai et al., 2021) that the vertebral arch closes at E18 in rats. As a ligament connecting adjacent vertebral arches, LF may experience mechanical stress after its growth, which stimulates collagen expression and promotes development (Cao et al., 2016).

At P0, the cells and fibers of MDB become orderly arranged. This may be due to the thin and loose muscle fibers of the embryonic rectus capitis minor, which may be insufficient to generate enough tension. After birth, enhanced muscle development and contraction-induce traction likely promote MDB fiber maturation, indicating that muscle plays an important role in MDB formation.

Evidence shows that muscle influences tendon development; tendons degenerate without embryonic muscle activity (Chen and Galloway, 2014). MDB resembles tendons in both composition—primarily type I collagen (Magnusson et al., 2008), and function, transmitting force via muscle contraction (Wei, 2005). Thus, MDB development is likely muscle dependent, similar to tendons. Supporting this, knockdown of the muscle-tendon junction specific gene *Col22a1* gene delays myodural bridge development in *Xenopus laevis* (Yang, 2023), confirming MDB as a tendinous structure influenced by muscle. Additionally, after P7, LF fibers exhibit increased curling, which may compensate for the reduced fiber numbers by filling inter-fiber spaces (Brown et al., 2012).

#### 4.1.2 Earlier structural maturation of MDB

Gomori aldehyde magenta staining revealed that the MDB consists of collagen fibers, while LF contains both elastic fibers and collagen fibers, consistent with previous studies (Nachemson and Evans, 1968; Lai et al., 2021). However, elastic fibers, the main component of LF, appeared only at P0, initially scattered and unbundled. In contrast, the MDB already exhibited a well-defined structure at P0, with subsequent fiber increase occurring without major morphological change. LF developed its mature structure later, which may correlate with delayed functional demand. Studies reported low elastin content in human fetal LF, gradually increasing during infancy toward adult levels (Song et al., 2000). Similarly, in bovine nuchal ligament, elastic fibers emerge in the second trimester (Ross and Bornstein, 1969), potentially due to early functional requirements (BROWN et al., 2012). Calves can stand and walk immediately after birth, whereas rats and humans, being altricial species, require prolonged development before bearing weight. As standing and walking begin, the need for spinal stability rises, enabling LF to fulfill its function.

In addition, elastic fibers development in perinatal rat lungs and aorta supports postnatal respiration circulation (Mariani et al., 2002; Kelleher et al., 2004). Thus, the appearance of elastic fibers enables structural maturation, providing the foundation for tissue function.

### 4.2 LOX/LOXL1 mediate fiber synthesis in MDB and LF

Histological results reveal distinct developmental processes and fiber composition between MDB and LF. These differences arise from molecular factors regulating fiber synthesis. This study confirms that LOX and LOXL1 mediate the fiber formation in both MDB and LF, providing a foundation for further investigation into their expression patterns during development.

LOX and LOXL isoenzymes differ from other copper amino oxidases in their quinone cofactor, the lysine tyrosylquinone (LTQ), which confers particular catalytic properties to these enzymes. BAPN is a specific and irreversible inhibitor of LOX activity that targets the active site of LOX and LOXL1 (Miana et al., 2015). To investigate the role of LOX and LOXL1 in MDB and LF development, we inhibited LOXL1 using BAPN, examined fiber changes via Masson staining, and assessed alterations in LOX and LOXL1 mRNA levels by RT-qPCR following enzymatic inhibition.

Masson staining revealed that inhibiting LOX and LOXL1 led to reduced fiber synthesis in both MDB and LF, resulting in sparser and abnormally distribution fibers. Prior studies showed that BAPN treatment increases collagen fiber spacing and decreases the mature-to-immature cross-linked ratio in osteoblasts (Canelón and Wallace, 2016), consistent with our histological findings. The results demonstrate that LOX and LOXL1 play important roles in the development of MDB and LF by mediating fiber synthesis.

RT-qPCR results revealed a compensatory increase in Lox and Loxl1 expression in both MDB and LF. Previous studies have shown that under low BAPN exposure, decreased Lox enzyme activity may trigger upregulation of LOX expression to restore normal enzyme levels (Canelón and Wallace, 2016), consistent with our observations. However, other studies report unchanged or reduced Lox expression, possibly due to higher BAPN doses exceeding compensatory capacity (Canelón and Wallace, 2016; Li et al., 2023). It is worth noting that if collagen and LOX expression are impaired at the DNA level, the inherent mechanical potential of the extracellular matrix may be inhibited, reducing LOX-mediated cell migration and ultimately hindering tissue repair (Brauer et al., 2023).

### 4.3 Divergent expression of LOX/LOXL1 in MDB vs. LF

Having confirming that LOX and LOXL1 mediate fiber synthesis in MDB and LF, this study examined their expression patterns during the development via immunohistochemistry. The peak expression of both enzymes coincided with the period of rising fiber synthesis. Furthermore, their postnatal upregulation corresponded to the stress-loading phase of MDB and LF.

#### 4.3.1 Peak LOX/LOXL1 expression synchronizes with fiber synthesis

This study examined LOX expression in MDB and LOX/LOXL1 expression in LF across developmental stages using immunohistochemistry and semi-quantitative analysis. Results indicated high LOX expression in MDB at E18, P7, and P21, while LOX and LOXL1 in LF were highly expressed during perinatal and postnatal stages. This peak expression coincided with increased fiber synthesis.

LOX expression in MDB was high at E18, P7, and P21. E18 coincides with the period of MDB formation in rats (Lai et al., 2021), during which increased fiber synthesis is required, consistent with the observed upregulation of LOX. By P7, pups can crawling, primarily driven by the head and forelimbs movement (Fox, 1965). Head motion induces contraction of the suboccipital muscles, exerting traction on MDB. Studies indicate that mechanical traction can promote fiber development, reflected by elevated LOX and collagen production (Chen et al., 2013). Thus, the high LOX expression at P7 in MDB may result from traction induced by contraction of the rectus capitis minor during crawling, facilitating MDB fiber synthesis. At P21, a significantly increase in MDB fiber quantity was observed, corresponding to the rise in LOX expression.

LOX and LOXL1 in LF are highly expressed mainly during the perinatal and postnatal period, with LOX expression slightly higher overall than LOXL1. Gomori aldehyde fuchsin staining revealed that elastic fibers in LF emerged at P0 and subsequently increased in density and thickness; This developmental process coincided with the peak expression of LOX and LOXL1, consistent with previously reported LOX expression patterns in mouse (Brown et al., 2012).

#### 4.3.2 Complementary LOX expression pattern aligns with mechanical stress periods

This study also compared LOX expression between MDB and LF during the same development stages. Results showed higher LOX expression in MDB than in LF at E17, E18, and P7, whereas LF exhibited higher expression at E20 and P14. The complementary expression pattern suggests that the postnatal peak in LOX expression corresponds to the period of mechanical stress in both MDB and LF.

At P7, when rats begin to crawl with frequent head movement and the abdomen still in contact with the ground, spine load is minimal and traction is primarily generated by the head (Fox, 1965). This head motion triggers contraction of the deep suboccipital muscles, exerting mechanical stress on MDB and coinciding with high LOX expressed. At P14, rats open their eyes, exhibit exploratory behavior (Goldey and Taylor, 1992), and start to run and jump with their abdomens lifted off the ground. Increased motor complexity and maturation of spinal column tissues at this stage straighten the fibers of the LF (Brown et al., 2012). This indicates that physical activity stretches the LF, establishing P14 as its main stress period, which also corresponds to elevated LOX expression.

The consistency between the peak LOX expression period in MDB/LF and the postnatal stress period may result from mechanical stretching caused by muscle contraction or spinal movement. Such stretching can activate integrins bound to collagen and fibronectin, triggering the release of TGF-β (transforming growth factor-β) and activating the TGF-β/Smad signaling pathway (Garamszegi et al., 2009). Within this pathway, phosphorylated Smad2/3 forms a complex with Smad4, which translocates into the nucleus and binds to the *Lox* gene promoter, enhancing its transcription and increasing fiber synthesis (Zhong et al., 2022). Thus, this study speculates that mechanical stretching of MDB or LF upregulates LOX expression, thereby promoting fiber production and tissue development. This proposed mechanism still requires further experimental verification.

### 4.4 Differential LOX/LOXL1 expression determines final MDB/LF structure

MDB and LF, both connective tissue originating from the mesoderm (Wachtler et al., 1981), differentiate under the guidance of specific transcription factors or signaling molecules that direct mesenchymal stem cells toward distinct fates (Nassari et al., 2017). Although LOX and LOXL1 act as downstream fiber synthesis factors (Miana et al., 2015), they are regulated by upstream cues that spatially and temporally separate the development of MDB and LF, ultimately leading to their structural differences in the posterior cervical region. Thus, while LOX and LOXL1 contribute to fiber synthesis, the final step in the formation, they along cannot be fully account for the divergent morphogenesis of MDB and LF.

Future studies should aim to identify the specific differentiation factors governing MDB and LF development. It may be useful to consider whether their differentiation parallels that of tendons and ligaments. Current research has revealed shared molecular markers between tendons and ligaments (Benjamin and Ralphs, 2000), yet genome-wide studies also demonstrate distinct transcriptional profiles (Pearse et al., 2009). Key regulators such as *Scx* (*Scleraxis,* specific to tendon stem cells) (Schweitze et al., 2001), along with *Mkx* (*Mohawk*), *Egr1 (Early Growth Response1)* and the TGF-β signaling pathway are known to regulate tendon differentiation (Ito et al., 2010; Lejard et al., 2011; Yu et al., 2022). Given that MDB, a tendon like structure connecting muscle to dura mater, is also composed primarily of collagen, transmits muscular tension, and also regulated by a TGF-β-centered signaling molecular network (Yang, 2023). it may share differentiation mechanisms with tendons. Furthermore, since MDB contains only collagen fibers and no elastin, its fibroblasts may suppress elastin gene expression. Transcription factors such as Fra-1 and JUN, which inhibit elastin transcription (Procknow and Kozel, 2022), could be involved and warrant investigation.

In this study, by examining SD rat embryos and newborn rats, the histological differences between MDB and LF were clarified. The synthesis of fibers in MDB and LF was confirmed by LOX and LOXL1, and the expression differences of LOX and LOXL1 in MDB and LF were explored, providing clues for the molecular regulation of MDB.

This study highlights the roles of LOX and LOXL1 in the final fiber assembly stage of MDB and LF, advances our understanding of MDB development, and offers new insights into its regulatory mechanisms. Limitations include the lack of detection of uncross-linked procollagen and tropoelastin after cross-linking, and insufficient exploration of the factors determining MDB and LF differentiation pathways. Nevertheless, the findings of this study establish a foundation for studying the initiation and regulation of differential differentiation in MDB.

## 5 Conclusion

① The MDB and LF exhibit distinct histological developmental patterns: cells and fibers in the MDB align in an organized manner later than those in the LF, while the MDB forms its complete structures earlier than the LF. ② LOX and LOXL1 play critical roles in collagen and elastic fibers formation, mediating fiber synthesis in both the MDB and LF. ③ Differential expression of LOX and LOXL1 during development leads to variations in fiber composition and maturation timing between the MDB and LF, contributing to their structural differences in the posterior cervical spinal interspace.

## Data Availability

The original contributions presented in the study are included in the article/supplementary material, further inquiries can be directed to the corresponding authors.
